# Causal links indicating ecosystem functioning in food webs

**DOI:** 10.1007/s42974-025-00267-0

**Published:** 2025-08-19

**Authors:** András Hidas, Ferenc Jordán

**Affiliations:** 1 Doctoral School of Environmental Sciences, ELTE Eötvös Loránd University, Budapest, Hungary; 2 Institute of Aquatic Ecology, HUN-REN Centre for Ecological Research, Budapest, Hungary; 3 KeyNode Research Ltd, Budapest, Hungary; 4 Stazione Zoologica Anton Dohrn, Naples, Italy; 5 Institute of Biological Research Cluj Subsidiary, National Institute of Research and Development for Biological Sciences, Bucharest, Romania

**Keywords:** Food web, Asymmetric interactions, Total biomass, Topology

## Abstract

Understanding the complexity of food webs is crucial for assessing ecosystem dynamics. However, the high number of trophic interactions in typical food webs complicates the identification of clear top-down or bottom-up regulatory effects. In this study, we applied interaction asymmetry analysis to examine causality within food webs, allowing for the identification of critical interactions based on topological importance (TI) metrics, which incorporate indirect interactions. We evaluated this method using 34 food web models from the Ecobase database and compared its performance with widely used network metrics. By constructing asymmetry graphs, we transformed original, undirected binary food webs into directed networks highlighting strong causal interactions. Pairwise correlation analyses revealed that ecosystems with higher total biomass indicated stronger bottom-up causal links and greater consumer diversity. Our findings suggest that asymmetry-based metrics provide robust quantitative indicators of causality, offering a straightforward yet powerful tool for assessing ecosystem functioning and health.

**Supplementary Information:**

The online version contains supplementary material available at 10.1007/s42974-025-00267-0.

## Introduction

In networks of interspecific interactions, all species can influence one another via direct interactions and indirect chain effects (Wootton, 1994). The principle “everything is connected to everything else,” although theoretically valid, does not facilitate understanding complexity. Beyond studies on non-local coherence (Laszlo, 2004) and indirect determination (Patten, 1991), network algebra attempts precise analysis and quantitative predictions on network effects. However, as trophic effects propagate both bottom-up (food provision) and top-down (control), their signs are mixed—positive for bottom-up and negative for top-down. The multiplicity of direct and indirect, positive and negative, as well as weak and strong effects severely limits the predictability of food web models (Dambacher et al., 2003).

Since effects influencing species *i* may originate from multiple other species, and symmetrically, effects spreading from species *i* may influence several others, it is challenging to understand cause-effect relationships. In a richly connected network, effects do propagate in all directions, to various distances and mixed effects mean that it is hard to understand whether, ultimately, species *i* affects species *j* or species *j* affects species *i*. Yet, if the interaction between species *i* and *j* is highly asymmetric, one may expect effects spreading mostly in one direction. An approach has been suggested for identifying causal effects based on the asymmetry of *ij* and *ji* effects. If causality is clear (e.g., changes in species *i* will generate changes in species *j* but not vice versa), we can make better predictions about food web dynamics. This simplification aims to handle complexity, similar to network linearization (Whipple & Patten, 1993) or constructing dominator trees (Allesina & Bodini, 2004). This method has been applied to several datasets (Jordán et al., 2014, 2024a, 2024b). Here, we test it on a large set of food webs.

## Methods

### Data

We used food web models from EcoBase (Colléter et al., 2015; Heymans et al., 2014). These models were filtered. First, only food webs with more than 50 nodes were kept. This criterion allowed us to exclude smaller food webs, which are less suitable for robust testing and analysis. Second, when an ecosystem was described across multiple years, only 1 year was retained to avoid overrepresentation. Consequently, models 768, 105, 107, 674, 400, 153, 755, and 526 were discarded based on the model description of the metadata. Finally, 34 food web models remained for analysis, treated as binary and undirected networks. The metadata of the used food web models can be found in Supplementary Table S1.

### The asymmetry graph

Using the *TI*^*n*^ index for indirect effects (up to *n* steps), one can calculate the strength of *ij* effects in the food web (Jordán, 2009). From the matrix of effects, the asymmetry between *ij* and *ji* effects can be calculated as

$${\varvec{A}}{ } = { }\left| {{\varvec{TI}}_{ij}^{3} - { }{\varvec{TI}}_{ji}^{3} } \right|$$

(Jordán et al., 2024a). Applying an appropriate threshold *t* identifies strongly asymmetric effects. These links and their endpoints compose the asymmetry graph. The asymmetry graph often includes a subset of the original food web links, although indirect effects can occasionally be strongly asymmetric. Constructing the asymmetry graph allows focusing on the predictable core of interspecific effects.

Although food webs are undirected and unweighted networks, the asymmetry graph is directed and weighted. Merely considering the topology of symmetric, binary interactions generates asymmetric and directed causal effects.

We used normalized values for *n* = 3 (*TI*^*3*^), with the threshold *t* set at the top 1% of all possible interactions. Thus, for a network with *N* nodes, we defined asymmetry graph links as the *N*(*N*-1)/2/100 most asymmetric effects. This represents 1% of every pair of nodes. *TI*^*3*^ serves as a topological index for keystone species assessment by incorporating indirect interactions over three steps. As argued in Jordán (2009), three steps capture the majority of ecologically meaningful effects while maintaining computational feasibility—a trade-off that appears optimal given the decaying influence of longer interaction chains. We conducted sensitivity tests by applying multiple thresholds for asymmetric effects (0.5%, 1%, 5%, 10%, and 20%). The 1% threshold yielded interactions that were most ecologically meaningful for the food webs analyzed. For smaller networks, however, a higher cutoff may be necessary to capture sufficient asymmetry signals. We note that alternative methods for studying causality also exist (Amaral-Zettler et al., 2021; Taruttis et al., 2015). Taruttis et al. combine conditional-independence screening with perturbation-driven structural equation models to infer directed edges in high-dimensional gene networks, whereas Amaral-Zettler et al. employ DAG-guided generalized propensity scores and distributed-lag nonlinear models to estimate continuous pollution impacts on health. In contrast, our approach infers interaction asymmetry in food webs directly from the network topology.

**Fig. 1:**
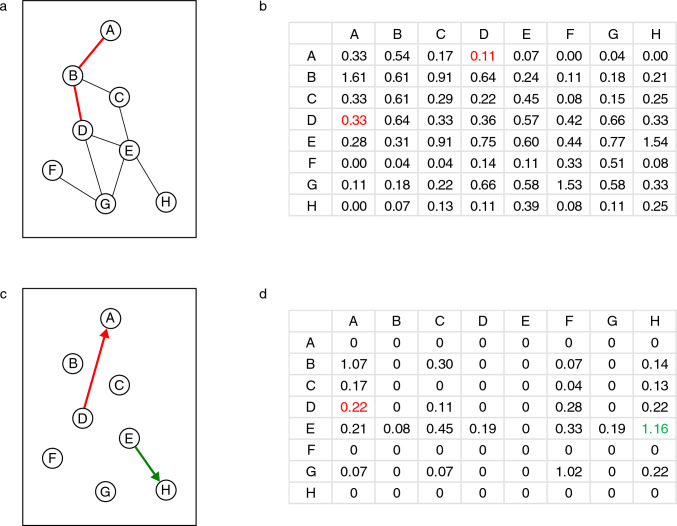
A toy network illustrating the relationship between the food web and the asymmetry graph. The food web is composed of eight nodes and nine interactions (**a**). According to the TI index, the matrix of *ij* effects is shown in **b** (species in row *i* affects species in column *j*). The two-step long indirect effect from species D to species A and the reverse effect from species A to species D are marked by red (also in a). The asymmetry values for *ij* and *ji* effects are shown in d (in red from D to A). The strongest asymmetry is shown in green; this effect composes the asymmetry graph (**c**). In this small network, the strongest 1% of all potential *N*(*N*-1)/2 asymmetries is only a single effect (rounded up), while in larger networks the number of links is larger in the asymmetry graph. Note that the original food web was undirected and unweighted but, based only on topology, the asymmetry graph is directed and weighted

**Fig. 2:**
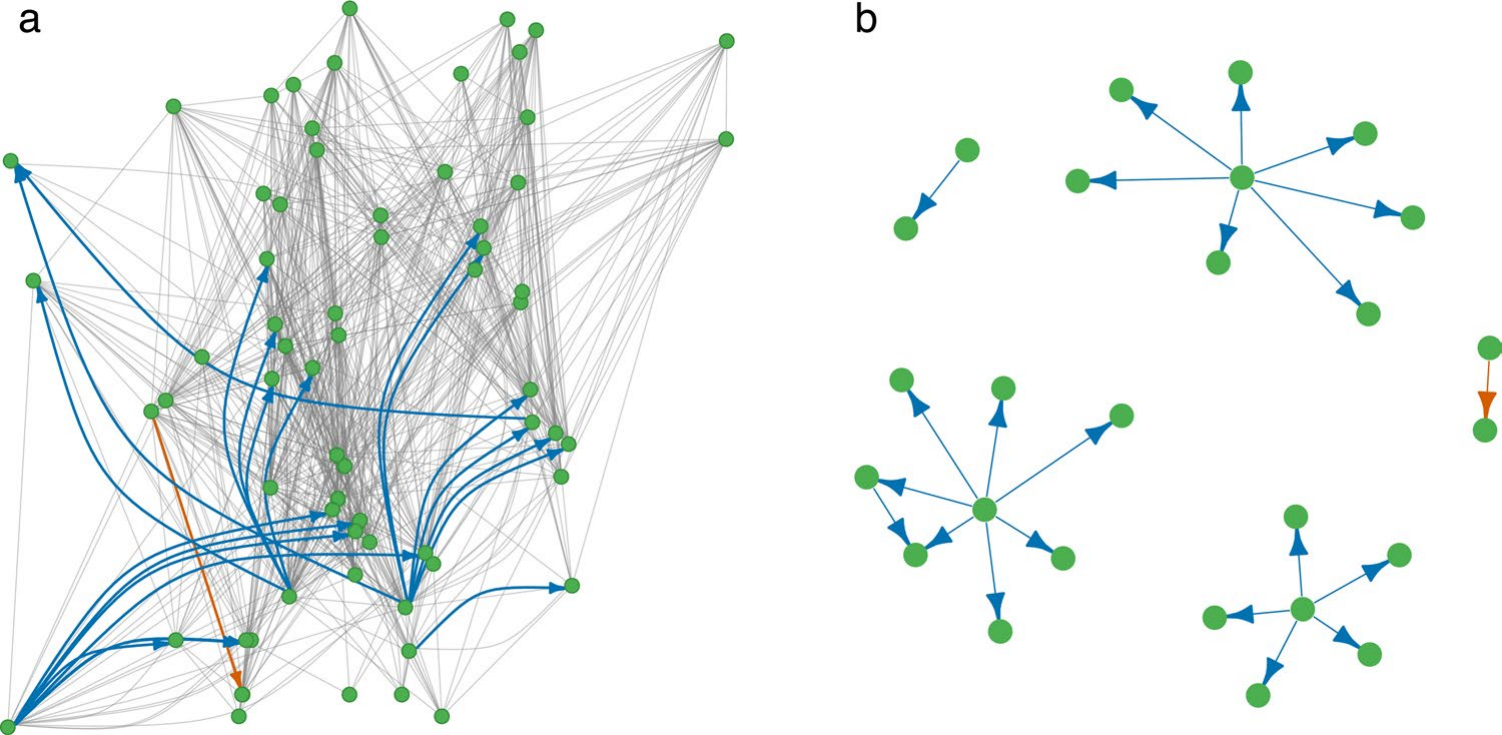
The food web with superimposed asymmetry graph highlighting directional relationships (**a**) and the isolated asymmetry graph (**b**) for model 765. Bottom-up effects are represented in blue, while top-down effects are represented in vermillion. Among the 708 original links, 22 asymmetric interactions (~ 3%) were identified

Figure 1 illustrates the relationship between the food web and asymmetry graph using a toy network. Figure 2 shows a typical food web (model 765) and its asymmetry graph with 5 components and 26 nodes. The food web and the asymmetry graph contain 67 and 26 nodes, respectively.

### Systemic indicators

We examined eight topological properties of food webs, eight properties of asymmetry graphs, two combined properties of food webs and asymmetry graphs, and three network-independent ecosystem functioning measures. These systemic indicators are widely used in food web research as general metrics for assessing structural and functional characteristics of ecosystems.

Food web properties include number of nodes (Nfw), number of links (Lfw), connectance (Cfw), average distance (avDISTfw), transitivity (Tfw), average trophic level (avTLfw), maximum trophic level (maxTLfw), and the ratio of species with TL > 2 (NCARNfw/Nfw; carnivores or omnivores, excluding producers and herbivores).

Asymmetry graph properties are the number of nodes (Nag), the number of links (Lag), the number of components (COMPag), the number of bottom-up effects (BUag), the number of top-down effects (TDag), their ratio (TDag/BUag), the number of source species with only outward effects (Nsoag), and the number of sink species with only inward effects (Nsiag). We also assessed whether asymmetry graphs contained directed cycles or were directed acyclic graphs (DAGs). All were DAGs, hence not used further in analysis. The ratio of nodes and links in the asymmetry graphs relates to the ratio of nodes and links in the food webs (Nag/Nfw and Lag/Lfw, respectively).

Network-independent (non-network) measures characterizing community structure and function include Shannon’s diversity index (Sh), total biomass (TB), and average ecotrophic efficiency (EE). Two unrealistic EE values were omitted for model 447.

### Statistical analysis

We investigated correlations between asymmetry graph properties, food web properties, and network-independent metrics. Our goal was to identify new information from asymmetry graphs as potential ecosystem-level indicators. We conducted pairwise Kendall’s τ correlation analysis with Benjamini and Hochberg p-value adjustment at p < 0.05 to control the false discovery rate (FDR) (Benjamini & Hochberg, 1995). We explicitly report that only correlations with adjusted p-values (q-values) below 0.05 were considered significant.

The TI indices, asymmetry values, and asymmetry graphs were computed using custom R functions, which were subsequently integrated into a new package called topoWeb. All analyses were performed using R Statistical Software v4.3.1 (R Core Team, 2023). Most network indices were computed with the igraph package (Csárdi et al., 2023), while correlations were assessed using the cor.test() base R function. Correlation plots were generated with the corrplot package (Wei & Simko, 2021).

## Results

**Fig. 3:**
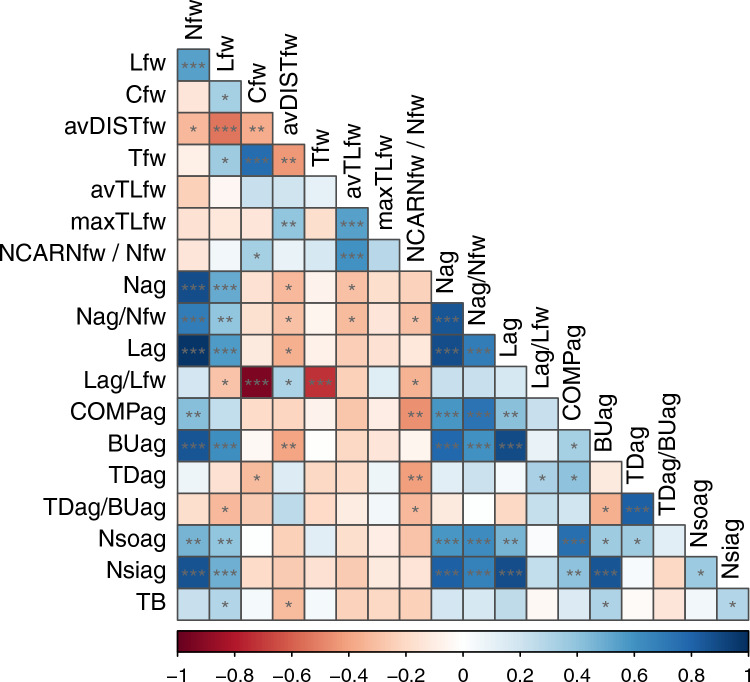
The correlation heatmap for the variables shown in Supplementary Table S3 (*: *p* < 0.05; **: *p* < 0.01; ***: *p* < 0.001)

Supplementary Table S2 presents all measured values from food web models. Statistical outcomes appear in Supplementary Table S3 and Fig. 3.

Various network measures were interrelated, reflecting mathematical dependencies. The strong correlation between Nfw and Lag is a mathematical artifact, as Lag is calculated from Nfw. Similarly, a higher maxTLfw inherently increases NCARNfw/Nfw.

However, some correlations among structural network measures are genuine. Higher food web connectance (Cfw) correlates with more top-down effects (TDag), indicating dense networks possess more top-down causalities.

We sought correlations between structural and functional variables. Ecotrophic efficiency (EE) and Shannon’s diversity index (Sh) showed no significant correlations with network properties. Total biomass (TB) correlated positively with link number (Lfw) and negatively with average distance (avDISTfw) in food webs. In asymmetry graphs, both bottom-up links (BUag) and sink numbers (Nsiag) positively correlated with TB.

## Discussion

Understanding complexity benefits from simple systemic indicators. We examined the asymmetry of interspecific dependencies using a large food web database. Our results indicate that the number of links in the food web (Lfw), bottom-up causalities (BUag), and the number of sink nodes (Nsiag) positively correlate with ecosystem total biomass (TB), while average distance in the food web (avDISTfw) correlates negatively. These are possible if the well-connected food web has more species at lower levels and only a few higher-level species: in this case, high asymmetries will emerge between the poorly connected higher level and the richly connected lower-level species, with asymmetric links directed in bottom-up direction.

Given the plausibility of these findings, we suggest these network properties as simple, quantitative indicators of ecosystem functioning. Extreme values in BUag or Nsiag may indicate unusual ecosystem functioning.

This approach shows promise for ecosystem monitoring and management by reducing food web complexity and isolating key asymmetric interactions. In addition, temporal changes in the asymmetry graph could serve as early-warning signals of shifts in community dynamics.

Future research should (1) explore threshold sensitivity (preliminary analyses indicated similar qualitative outcomes across thresholds), (2) compare causality approaches (e.g., our method vs. Amaral-Zettler et al. (2021)), (3) evaluate directed and weighted food webs (calculating *WI*^*n*^ instead of *TI*^*n*^), and (4) to study also the identity of species in the asymmetry graphs (frequent species, sinks, and sources).

## Supplementary Information

Below is the link to the electronic supplementary material.

Supplementary file1 Supplementary Table S1 contains the metadata of the used Ecobase food web models including references as queried directly from the database.

Supplementary file2 Supplementary Table S2 contains the 21 variables studied for the 34 food webs.

Supplementary file3 Supplementary Table S3 contains Kendall’s τ correlation coefficients among 21 variables from Supplementary Table S2.

## Data Availability

We used data for our study from Ecobase which is an open-access database. The reference of each original food web we used is listed in Supplementary Table S1. The description of the database can be found here: https://ecobase.ecopath.org.The codes to calculate the TI index and the asymmetry were bundled to an R package called topoWeb. You can download this package from https://github.com/hidasandris/topoweb.
